# Risk of Malignant Neoplasia with Glucagon-Like Peptide-1 Receptor Agonist Treatment in Patients with Type 2 Diabetes: A Meta-Analysis

**DOI:** 10.1155/2019/1534365

**Published:** 2019-07-16

**Authors:** Yufang Liu, Xiaomei Zhang, Sanbao Chai, Xin Zhao, Linong Ji

**Affiliations:** ^1^Department of Endocrinology, Peking University International Hospital, Beijing 102206, China; ^2^Department of Endocrinology, Peking University People's Hospital, Beijing 100044, China

## Abstract

**Background:**

Glucagon-like peptide-1 (GLP-1) receptor agonists are effective glucose-lowering drugs, but there is concern that they may increase the risk of malignant neoplasia. The present meta-analysis examined the safety of GLP-1 receptor agonists with regard to malignant neoplasia.

**Methods:**

We analyzed data from randomized controlled trials with a minimum duration of 24 weeks that assessed the incidence of neoplasms in type 2 diabetes patients receiving GLP-1 receptor agonists compared with placebo or other hypoglycemic drugs. We searched the MEDLINE, Embase, and Cochrane databases with a language restriction of English through October 1, 2018, and carried out a meta-analysis of the available trial data using a fixed effects model to calculate odds ratios (ORs) for neoplasia.

**Results:**

Thirty-four relevant articles, providing data for 50452 patients, were included in the meta-analysis. Compared with the incidence of malignant neoplasia with placebo or other interventions, no increase in malignant neoplasm formation was observed with the use of GLP-1 receptor agonists (OR 1.04, 95% confidence interval (CI) 0.94–1.15; *p* = 0.46), liraglutide (OR 1.08, 95% CI 0.91–1.27; *p* = 0.38), exenatide (OR 1.00, 95% CI 0.86–1.16; *p* = 1.00), semaglutide (OR 0.89, 95% CI 0.35–2.22; *p* = 0.80), or albiglutide (OR 1.07, 95% CI 0.23–4.88; *p* = 0.93). A subanalysis of trials lasting longer than 3 years also showed no increase in the neoplasia risk with GLP-1 receptor agonist use (OR 1.03, 95% CI 0.92–1.15; *p* = 0.60). Between-trial statistical heterogeneity was low for all comparisons.

**Conclusion:**

GLP-1 receptor agonists can be used without safety concerns related to malignant neoplasia in patients with type 2 diabetes.

## 1. Introduction

Glucagon-like peptide-1 (GLP-1) receptor agonists are a class of hypodermic hypoglycemic drugs that are effective for the treatment of type 2 diabetes mellitus. A number of GLP-1 receptor agonists have already been approved by the Food and Drug Administration (FDA) for type 2 diabetes treatment, including exenatide, liraglutide, semaglutide, lixisenatide, dulaglutide, albiglutide, and others. Based on the findings of several cardiovascular outcome trials (CVOTs) [1], GLP-1 receptor agonists and sodium-glucose cotransporter 2 (SGLT2) inhibitors are highly recommended as types of antidiabetic drugs, second only to metformin. GLP-1 receptor agonists are especially recommended, given that cardiovascular disease is the major cause of death among patients with type 2 diabetes.

However, some studies have indicated that GLP-1 receptor agonists may be associated with an increased risk of malignant neoplasia. In animal models, GLP-1 receptor agonist treatment was linked to an increased risk of pancreatic cancer and thyroid C-cell cancer [2, 3]. At the same time, studies conducted in humans found increased risks of acute pancreatitis and pancreatic cancer with the use of GLP-1 receptor agonists [4]. In 2014, a US FDA and European Medical Association (EMA) assessment published in the New England Journal of Medicine stated that a final conclusion could not be made regarding a causal relationship between incretin-based drugs and pancreatitis or pancreatic cancer. Thus, additional meta-analyses and continued investigations into the safety of these drugs are needed [5]. Since then, several reviews have explored the associations between incretin-based drugs and pancreatic cancer [6–10]. Until now though, almost all reviews have focused on pancreatic cancer, and no review studying the association of GLP-1 receptor agonists with all types of malignant neoplasms has been published, despite the availability of data for the incidence of various types of cancer, including breast cancer, prostate cancer, and others, in patients taking GLP-1 receptor agonists. Therefore, the objective of this meta-analysis was to summarize the evidence for an association between GLP-1 receptor agonists and the incidence of all forms of malignant neoplasms.

## 2. Methods

This systematic review was registered in PROSPERO (registration number CRD42019122052).

### 2.1. Search Strategy and Study Selection Criteria

In this systematic review and meta-analysis, we regarded studies as eligible for the inclusion criteria if they were randomized controlled trials (RCTs) that included adult patients with type 2 diabetes, compared a GLP-1 receptor agonist to another treatment strategy with a minimum treatment duration of 24 weeks, and reported the number of participants who developed neoplasms during follow-up. We retained all potentially eligible studies for review, independent of the primary outcome of each study. We searched the MEDLINE, Embase, and Cochrane databases for eligible trials, with a language restriction of English. The search strategy was based on “subject terms+free terms.” Subject terms used in the searches were “glucagon-like peptide-1 receptor agonist,” “exenatide,” “liraglutide,” “semaglutide,” “lixisenatide,” “dulaglutide,” “albiglutide,” “neoplasms,” and “diabetes mellitus.” With regard to neoplasms, we included studies of all types of malignant tumors and excluded those evaluating benign tumor formation. For the search for RCTs, we used available filters to search only for RCTs from the Harvard Library. Searches were done through October 1, 2018.

Two independent investigators reviewed study titles and abstracts, and studies that satisfied the inclusion criteria were assessed by screening of the full text. Trials selected for detailed analysis and data extraction were analyzed by two investigators, and disagreements were resolved by a third investigator. For quality assessment, Cochrane Collaboration's Tool for Assessing Risk of Bias in RCTs was used.

### 2.2. Statistical Analysis

When specific data were not available, requests for the information were sent to the corresponding authors of the trial articles. We calculated ORs and 95% confidence intervals (CIs) for the numbers of neoplasia events by the treatment group. We used a fixed effects model meta-analysis if the between-trial statistical heterogeneity was low. We used a random effects model meta-analysis if the between-trial statistical heterogeneity was high. The possibility of publication bias was assessed by constructing a funnel plot of each trial's effect size against the standard error. The heterogeneity of treatment effects between trials was assessed by the *I* ^2^ index and Cochran's *Q* test, with *p* values < 0.05 on Cochran's *Q* test representing significant heterogeneity. The *I* ^2^ index thresholds describing the degree of heterogeneity were 25% or lower (low), 26–50% (moderate), and greater than 50% (high). RevMan (version 5.1) software was used for all statistical analyses.

## 3. Results

### 3.1. Study Selection

**Figure 1:**
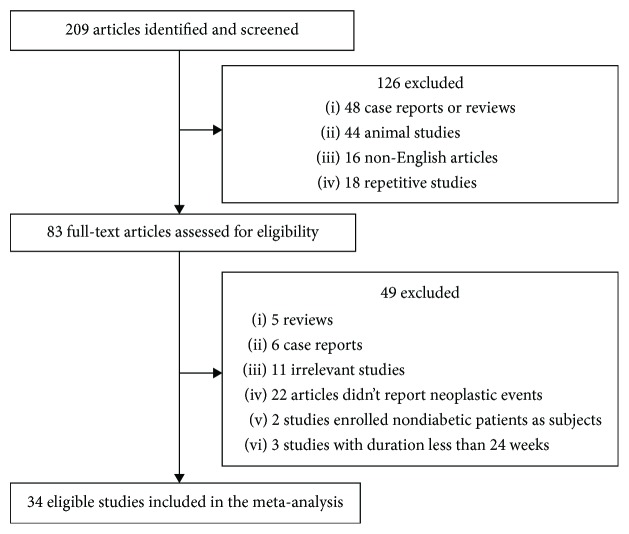
Study selection.

Our database searches identified 209 studies, of which 34 (presenting data for 50452 participants) were included in our analysis (Figure 1). Among the initial 209 trials, 126 were excluded for being a case report, review, animal study, non-English article, or repetitive study based on reading of the title and abstract. For the remaining 83 articles, two authors separately read the full-text articles in detail to assess their eligibility, and 49 trials were further excluded (5 reviews, 6 case reports, 11 irrelevant studies, 22 not reporting neoplastic events, 2 with nondiabetic subjects, and 3 with a duration less than 24 weeks). The 34 trials included in the final analyses were all published between 2010 and 2017 (Table 1). The trial duration ranges from 24 to 198 weeks, and all trials excluded patients with a history of neoplasms at baseline.

Fourteen trials compared treatment outcomes achieved with GLP-1 receptor agonists versus placebo with or without oral antidiabetic drugs. The other 20 included trials compared outcomes achieved with GLP-1 receptor agonists to those obtained with metformin (2 trials), sitagliptin (7 trials), glimepiride (1 trial), premixed insulin (1 trial), glargine (8 trials), or lispro (1 trial). Many types of neoplasms occurred in these trials, including pancreatic cancer, thyroid cancer, breast cancer, gastrointestinal cancer, skin cancer, leukemia, lymphoma, prostate cancer, and others. For trials belonging to the same program, such as the DURATION-2, DURATION-3, and DURATION-4 studies, detailed assessment was performed to exclude duplicate data.

### 3.2. Incidence of Neoplasia with All GLP-1 Receptor Agonists

**Figure 2:**
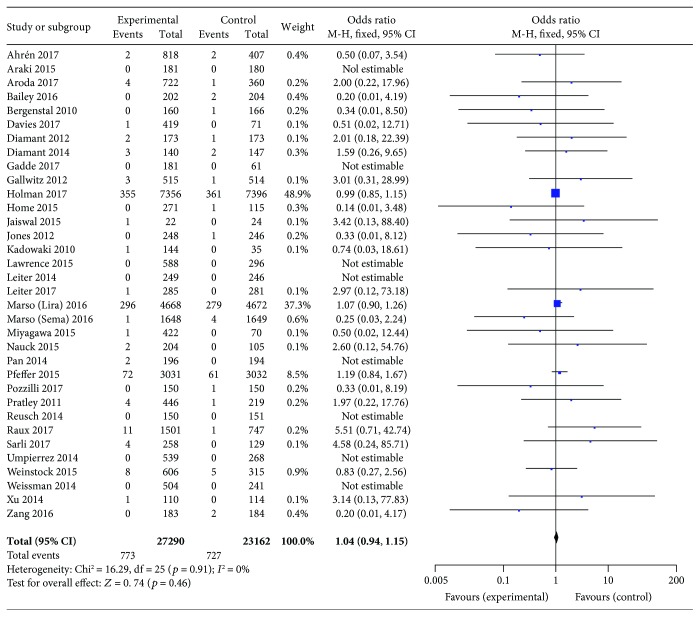
Meta-analysis of the incidence of neoplasms with the use of all tested GLP-1 receptor agonists versus placebo or other antidiabetic treatments.

**Figure 3:**
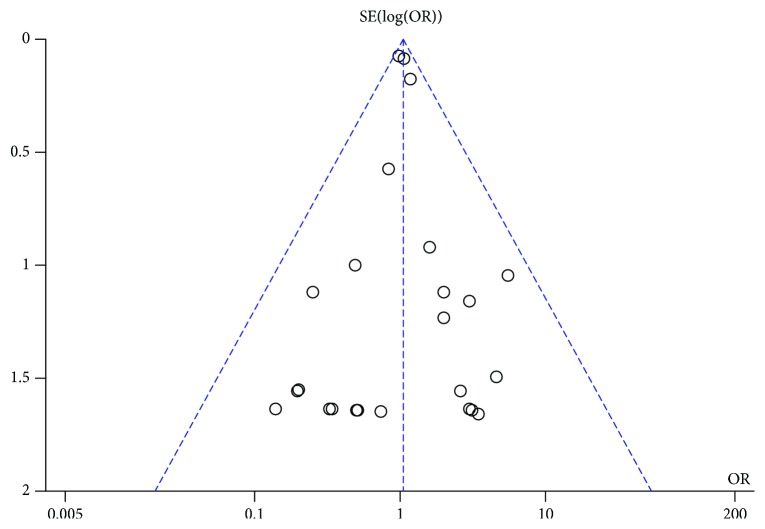
Funnel plot for the comparison of the incidence of neoplasia with the use of GLP-1 receptor agonists versus placebo or other antidiabetic treatments.

Compared with placebo or other interventions, GLP-1 receptor agonist use showed no association with an increased risk of neoplasm development (OR 1.04, 95% CI 0.94–1.15; *p* = 0.46), with no statistically significant between-study heterogeneity (*I* ^2^ = 0%, *p* = 0.91; Figure 2). Eight trials were not included in this analysis, because no neoplasms were reported among their patients. The funnel plot for this analysis indicated no significant publication bias (Figure 3).

### 3.3. Subgroup Analyses of the Incidence of Neoplasia with Different GLP-1 Receptor Agonists

**Figure 4:**
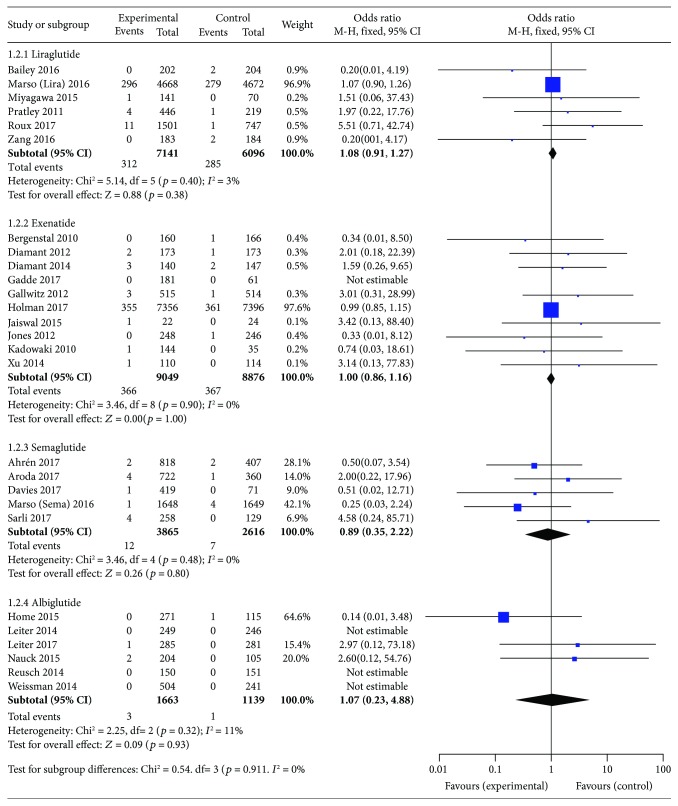
Meta-analysis of the incidence of neoplasms with the use of specific GLP-1 receptor agonists versus placebo or other antidiabetic treatments.

Among all 34 included trials, 6 trials (with data for 13237 patients) employed liraglutide as the experimental agent. Compared with placebo or other interventions, liraglutide use was not associated with an increased incidence of neoplasms (OR 1.08, 95% CI 0.91–1.27; *p* = 0.38), and no statistically significant between-study heterogeneity was observed (*I* ^2^ = 3%, *p* = 0.40; Figure 4).

Among all 34 included trials, 10 trials (with data for 17925 patients) employed exenatide as the experimental agent. Compared with placebo or other interventions, exenatide use was not associated with an increased incidence of neoplasia (OR 1.00, 95% CI 0.86–1.16; *p* = 1.00), and no statistically significant between-study heterogeneity was observed (*I* ^2^ = 0%, *p* = 0.90; Figure 4). One trial that used exenatide was excluded from the meta-analysis, because it did not report any neoplasia events.

Among the 34 included trials, 5 trials (with data for 6481 patients) employed semaglutide as the experimental agent. Compared with placebo or other interventions, semaglutide use was not associated with an increase in neoplasm formation (OR 0.89, 95% CI 0.35–2.22; *p* = 0.80), and no statistically significant between-study heterogeneity was observed (*I* ^2^ = 0%, *p* = 0.48; Figure 4).

Among the 34 included trials, 6 trials (with data for 2802 patients) employed albiglutide as the experimental agent. Compared with placebo or other interventions, albiglutide use was not associated with an increased incidence of neoplasia (OR 1.07, 95% CI 0.23–4.88; *p* = 0.93), and no statistically significant between-study heterogeneity was observed (*I* ^2^ = 11%, *p* = 0.32; Figure 4). Data from three trials were not included in this comparison, because they did not report any neoplasia events.

### 3.4. Incidence of Neoplasia with GLP-1 Receptor Agonists versus Placebo

**Figure 5:**
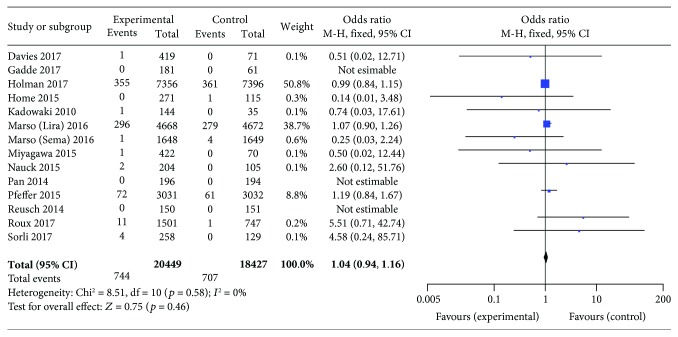
Meta-analysis of the incidence of neoplasia with placebo versus GLP-1 receptor agonists used.

Among the 34 included trials, 14 trials (with data for 38876 patients) chose placebo as the only control treatment. Compared with placebo only, GLP-1 receptor agonist use was not associated with an increased incidence of neoplasia (OR 1.04, 95% CI 0.94–1.16; *p* = 0.46), and no statistically significant between-study heterogeneity was observed (*I* ^2^ = 0%, *p* = 0.58; Figure 5). Data from three trials were not included in this comparison, because they did not report any neoplasia events.

### 3.5. Subanalysis including Only Trials Lasting at Least 3 Years

**Figure 6:**
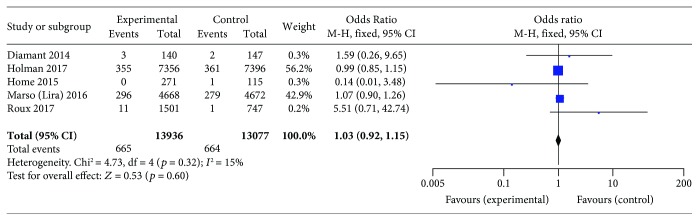
Meta-analysis of the incidence of neoplasia with the use of a GLP-1 receptor agonist versus placebo or other antidiabetic treatments, based on studies with a minimum duration of 3 years.

Among the 34 included trials, 5 trials (with data for 1309 patients) had a study duration of at least 3 years. The subanalysis of only these 5 trials showed that, compared with placebo or other antidiabetic treatments, GLP-1 receptor agonist use was not associated with an increased incidence of neoplasia (OR 1.03, 95% CI 0.92–1.15; *p* = 0.60), and no statistically significant between-study heterogeneity was observed (*I* ^2^ = 15%, *p* = 0.32; Figure 6).

## 4. Discussion

Previous studies have reported that GLP-1 receptor agonist use correlated with an increased risk of pancreatic cancer [4, 44]. Although consistent preclinical, pharmacovigilance, and epidemiologic evidence is lacking, considerable attention has been paid to the potential association between GLP-1 receptor agonists and pancreatic cancer [45–47]. Based on the results of animal studies [2, 44], researchers have speculated that chronic overstimulation of GLP-1 receptors in pancreatic cells could induce pancreatitis, ultimately leading to an increased risk of pancreatic cancer. This speculation has been supported by pharmacovigilance reports [4, 48], and animal studies have also suggested a higher incidence of thyroid C-cell adenomas and carcinomas with once-weekly exenatide than with placebo. Specifically, higher rates of thyroid C-cell tumors and hyperplasia were observed in rodents treated with liraglutide than in control animals [3]. However, these findings have not been replicated in humans.

GLP-1 receptor agonists promote cell proliferation and survival by activating signaling pathways in human islet cells, such as those involving phosphate idylinositol 3 kinase (PI3K) and extracellular regulated kinases 1 and 2 (ERK1/2), which are also frequently activated in human colon cancer cells. ERK1 and ERK2 act on transcription factors such as E1k-1, c-myc, c-fos, c-jun, activating transcription factor (ATF), nuclear factor- (NF-) kB, and activator protein- (AP-) 1, to promote the expression of genes closely related to cell proliferation and differentiation [49]. Thus, it is possible that GLP-1 receptor agonists promote the proliferation of cancer cells, and with the important clinical implications of such an effect, it is necessary to clarify the effects of GLP-1 receptor agonists on cancer cells.

Research about dipeptidyl peptidase-4 (DPP-4) inhibitors, which function via a similar mechanism as GLP-1 receptor agonists, failed to verify an association between the use of these drugs and an increased risk of site-specific cancer, and this was attributed to the small number of studies for each cancer type and their relatively short duration [50]. Another meta-analysis published in 2017 that included four large-scale studies indicated that GLP-1 receptor agonists did not increase the risk of pancreatic cancer [51], and our study further demonstrated that GLP-1 receptor agonist therapy was not associated with an increased risk of any of the malignant neoplasms studied. This result was true for GLP-1 receptor agonists overall as well as for the specific GLP-1 receptor agonists exenatide, liraglutide, semaglutide, dulaglutide, and albiglutide. Because only two trials used lixisenatide as the experimental intervention, we did not analyze the data for this drug separately, and this was also the case for dulaglutide. Because the between-trial statistical heterogeneity was low for all comparisons, sensitivity analysis was not conducted.

The main strength of this review is that all of the included studies were RCTs. Among all included trials, two RCTs contributed considerable weight to the pooled analysis [1, 19]. All of the included trials reported consistent results regarding the risk of neoplasms with GLP-1 receptor agonist use, and the meta-analysis strengthened the overall conclusion through the analysis of a much larger sample.

We excluded studies with an intervention duration less than 24 weeks to prevent detection bias or even reverse causality. According to the incidence rates of malignant tumors in humans [52], it is not likely that cancer diagnosed within 24 weeks after initiation of GLP-1 receptor agonist intervention is causally related to the experimental agent. Further analysis of trials with a duration of at least 3 years was conducted, as any increase in the incidence of neoplasms due to GLP-1 receptor agonist stimulation should be more readily detected in these trials.

A limitation of the present meta-analysis was the lack of trials with long-term duration, given that neoplasm formation may occur over an extended period. Additionally, patients included in RCTs are generally healthier than real-world patients and are therefore less likely to develop neoplasms than the general patient population or patients in observational studies of new drugs. Moreover, as the occurrence of neoplasia was not the primary or secondary outcome in these RCTs, reporting bias is possible. Although most of the included studies were published in high-impact journals, potential risks of bias such as an open-label design and funding from pharmaceutical companies are still possible, as outlined in the supplementary tables (supplementary material (available here)). Another limitation is that we could not make stratified comparisons according to the different types of neoplasm due to the paucity of original data. Finally, while we excluded studies with a duration less than 24 weeks, most studies did not report the time of neoplasm diagnosis after study enrollment. As a result, some patients likely were diagnosed with a neoplasm after receiving GLP-1 receptor agonist intervention for less than 24 weeks but could not be excluded.

## 5. Conclusion

GLP-1 receptor agonists can be used without safety concerns related to the risk of malignant neoplasia in patients with type 2 diabetes.

## Figures and Tables

**Table 1:** Trial design features and results.

Author/year	Trial/program	Interventions		Trial duration (weeks)	Experimental		Control	
		Experimental	Control		Number of events	*N*	Number of events	*N*
Gallwitz et al. 2012 [11]	EUREXA	Exenatide	Glimepiride	102	3	515	1	514
Russel-Jones et al. 2012 [12]	DURATION-4	Exenatide	Metformin	26	0	248	1	246
Kadowaki et al. 2010 [13]		Exenatide	Placebo	24	1	144	0	35
Xu et al. 2014 [14]	CONFIDENCE	Exenatide	Premixed insulin	48	1	110	0	114
Jaiswal et al. 2015 [15]		Exenatide	Glargine	77	1	22	0	24
Diamant et al. 2014 [16]	DURATION-3	Exenatide	Glargine	156	3	140	2	147
Diamant et al. 2012 [17]		Exenatide	Glargine	84	2	173	1	173
Bergenstal et al. 2010 [18]	DURATION-2	Exenatide	Sitagliptin	26	0	160	1	166
Holman et al. 2017 [19]	EXSCEL	Exenatide	Placebo	167	355	7356	361	7396
Gadde et al. 2017 [20]	DURATION-NEO-2	Exenatide	Placebo	28	0	181	0	61
Weinstock et al. 2015 [21]	AWARD-5	Dulaglutide	Sitagliptin	26	8	606	5	315
Araki et al. 2015 [22]		Dulaglutide	Glargine	26	0	181	0	180
Blonde et al. 2015 [23]	AWARD-4	Dulaglutide	Glargine	52	0	588	0	296
Umpierrez et al. 2014 [24]	AWARD-3	Dulaglutide	Metformin	52	0	539	0	268
Pozzilli et al. 2017 [25]	AWARD-9	Dulaglutide	Glargine	28	0	150	1	150
Miyagawa et al. 2015 [26]		Dulaglutide	Placebo	52	0	281	0	70
		Liraglutide			1	141		
Bailey et al. 2016 [27]	LIRA-SWITCH	Liraglutide	Sitagliptin	26	0	202	2	204
Zang et al. 2016 [28]		Liraglutide	Sitagliptin	26	0	183	2	184
Marso et al. 2016 (Liraglutide) [1]	LEADER	Liraglutide	Placebo	198	296	4668	279	4672
Pratley et al. 2011 [29]		Liraglutide	Sitagliptin	52	4	446	1	219
le et al. 2017 [30]	SCALE	Liraglutide	Placebo	160	11	1501	1	747
Marso et al. 2016 (Semaglutide) [31]	SUSTAIN-6	Semaglutide	Placebo	104	1	1648	4	1649
Ahrén et al. 2017 [32]	SUSTAIN 2	Semaglutide	Sitagliptin	52	2	818	2	407
Sorli et al. 2017 [33]	SUSTAIN 1	Semaglutide	Placebo	30	4	258	0	129
Davies et al. 2017 [34]		Semaglutide	Placebo	26	1	419	0	71
Aroda et al. 2017 [35]	SUSTAIN 4	Semaglutide	Glargine	30	4	722	1	360
Reusch et al. 2014 [36]	HARMONY 1	Albiglutide	Placebo	52	0	150	0	151
Home et al. 2015 [37]	HARMONY 5	Albiglutide	Placebo	156	0	271	1	115
Weissman et al. 2014 [38]	HARMONY 4	Albiglutide	Glargine	52	0	504	0	241
Nauck et al. 2015 [39]	HARMONY 2	Albiglutide	Placebo	52	2	204	0	105
Leiter et al. 2014 [40]		Albiglutide	Sitagliptin	52	0	249	0	246
Leiter et al. 2017 [41]		Albiglutide	Lispro	52	1	285	0	281
Yu et al. 2014 [42]	GetGoal-M-Asia	Lixisenatide	Placebo	24	0	196	0	194
Pfeffer et al. 2015 [43]	ELIXA	Lixisenatide	Placebo	108	72	3031	61	3032

## Supplementary Materials

Supplementary Materials The risk of bias graph review of authors' judgements about each risk of bias item presented as percentages across all included studies.
