# Supplementary figures and images for: Risk of Malignant Neoplasia with Glucagon-Like Peptide-1 Receptor Agonist Treatment in Patients with Type 2 Diabetes: A Meta-Analysis
Source: J Diabetes Res. 2019 Jul 16;2019:1534365. doi: 10.1155/2019/1534365 (PMC6664552; doi:10.1155/2019/1534365)

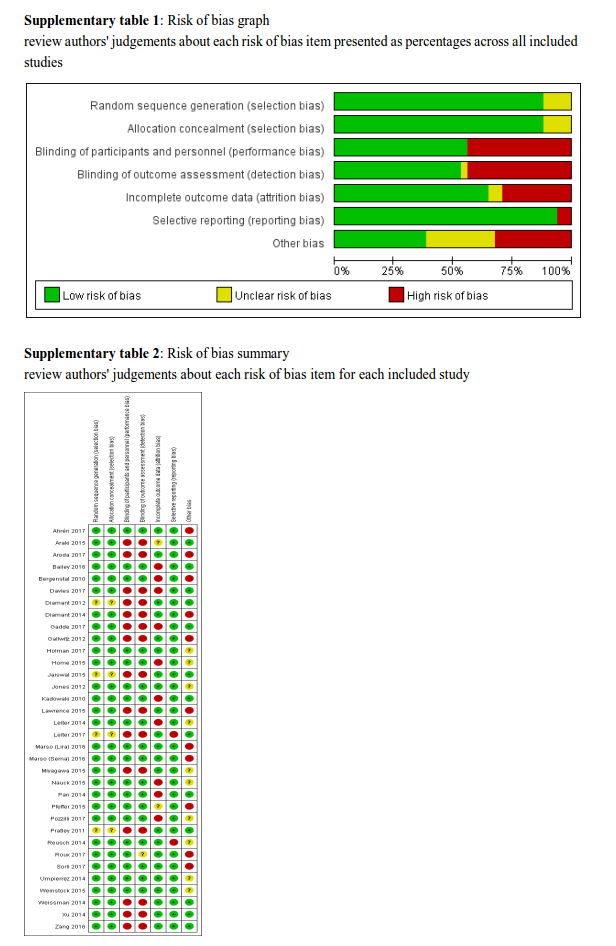

Supplement: Supplementary Materials — The risk of bias graph review of authors' judgements about each risk of bias item presented as percentages across all included studies. [file 1534365.f1.zip › mat.1534365.v2.docx]
